# The course and outcomes of complicated gallstone disease in pregnancy: Experience of a tertiary center

**DOI:** 10.4274/tjod.65475

**Published:** 2016-12-15

**Authors:** Mehmet İlhan, Gülşah İlhan, Ali Fuat Kaan Gök, Kayıhan Günay, Cemalettin Ertekin

**Affiliations:** 1 İstanbul University İstanbul Faculty of Medicine, Department of General Surgery, İstanbul, Turkey; 2 Süleymaniye Maternity and Child Diseases Training and Research Hospital, Clinic of Obstetrics and Gynecology, İstanbul, Turkey

**Keywords:** Acute cholecystitis, acute pancreatitis, cholangitis, choledocholithiasis, Pregnancy

## Abstract

**Objective:**

To evaluate the course and outcomes of pregnant patients with complicated gallstone disease and to reveal the experience of a tertiary center.

**Materials and Methods:**

The records of 92.567 patients were evaluated using searches for diagnoses with the terms of pregnant, pregnancy, gallstone, cholecystitis, cholangitis, choledocholithiasis, pancreatitis, and endoscopic retrograde cholangiopancreatography in pregnancy in the hospital database. Patients’ age, week of gestation, parity, body mass index, definitive diagnosis, attack episodes, treatment modalities, and obstetric and neonatal complications were evaluated.

**Results:**

Overall, 59 women were diagnosed as having complicated gallstone disease in pregnancy. Acute cholecystitis was the most commonly diagnosed complicated gallbladder disease (62.7%). Cholecystectomy was performed in 15 women during gestation. Perinatal outcomes were as follows: one (1.7%) maternal death, 4 (6.8%) preterm deliveries, 5 (8.5%) low-birth-weight fetuses, and 1 (1.7%) missed abortion were encountered. No fetal abnormalities were encountered.

**Conclusion:**

A significant proportion of women experience biliary disease during pregnancy. Herein, we presented our clinical experience because the diagnosis, course, and management of complicated gallstone disease in pregnancy is complicated.

## INTRODUCTION

Gallstone disease is a common reason for non-gynecologic operations during pregnancy^(1,2)^ and is the major non-obstetric cause for hospitalization in the first year postpartum^(3)^. A significant proportion of women experience biliary disease during pregnancy. Pregnancy may accentuate gallbladder stone formation. Alterations in hepatobiliary function occur during pregnancy to create a lithogenic environment. These changes include secretion of bile with increased amounts of cholesterol and decreased amounts of chenodeoxycholic acid and gallbladder stasis^(4)^. The prevalence of biliary sludge, gallstones, and biliary pancreatitis in pregnancy ranges from 5-36%, 2-11%, and 1/1000-3/10000, respectively^(1,5,6,7)^. In addition to the risks of symptomatic biliary disease on the mother and fetus, treatment approaches including surgery and conservative treatment bring their own risks and restrictions in pregnancy. Though recent guidelines have recommended laparoscopic cholecystectomy (LC) during pregnancy for all symptomatic gallstone disease^(8,9)^, management of symptomatic disease during pregnancy has often been nonsurgical to avoid fetal and maternal harm^(10)^. However, this non-operative management leads to a very high rate of antepartum symptom recurrence^(2,8)^. The aim of the present study was to determine the course and outcomes of pregnant patients with complicated gallstone disease and to reveal the experience of a tertiary center.

## MATERIALS AND METHODS

The written and electronic medical records of 92.567 patients who were admitted to the Emergency Surgery Clinic in İstanbul University İstanbul Faculty of Medicine, between January 2010 and August 2015, were evaluated in this study. The medical records were reviewed using searches for diagnoses with the terms of pregnant, pregnancy, gallstone, cholecystitis, cholangitis, choledocholithiasis, pancreatitis, and endoscopic retrograde cholangiopancreatography (ERCP) in pregnancy in the hospital database. Patients diagnosed as having complicated gallstone disease were included in this study. Complicated gallstone disease was defined as acute cholecystitis, choledocholithiasis, cholangitis, and gallstone pancreatitis^(3)^. All diagnoses were made with a combination of medical history, physical examination, laboratory tests and imaging techniques such as [ultrasonography, and magnetic resonance cholangiopancreatography (MRCP)]. Diagnosis of acute cholecystitis was made when biliary pain was associated with the presence of both gallbladder lithiasis and inflammation. Acute biliary pancreatitis was diagnosed with the presence of gallbladder lithiasis, elevated serum amylase level, and presence of biliary pain. Choledocholithiasis was defined in the presence biliary symptoms, jaundice, abnormal liver function tests, and presence of gallbladder lithiasis. Common bile duct stones were confirmed with either ultrasonography or MRCP; these patients underwent ERCP. Cholangitis was diagnosed in the presence of fever and elevated acute phase reactants. LC and ERCP procedures were performed after explanation of the risks, complications, and alternatives. ERCPs were performed by general surgeons who were experienced with endoscopy using a Fujinon EPX-201 videoendoscope. Double-sided lead shielding was positioned above, below, and on both sides of the patient, covering the abdomen and pelvis in case there was a need for radiation. In all cases, selective cannulation was performed and confirmed by the aspiration and/or direct visualization of the bile. After cannulation of the common bile duct, a guide wire was passed and sphincterotomy was completed. Stones were extracted using a basket or balloon sweep^(11)^. LC was performed using a standard four-port technique. A hasson trocar was placed, and the abdominal cavity was insufflated with carbon dioxide, with a maximum insufflation pressure of 12 mmHg. Calot’s triangle was identified and the cystic duct and cystic artery were clipped, taking care not to injure the common bile duct. The gallbladder was removed from the liver bed using diathermy. If clear exploration could not be provided using laparoscopy, laparotomic cholecystectomy was performed. Patients with pregnancy-related conditions that may be associated with epigastric pain including severe preeclampsia, hemolysis, elevated liver enzymes, and low platelet count syndrome, acute fatty liver, abruptio placentae, uterine rupture, and intraamniotic infection, and patients with primary sclerosing cholangitis, non-biliary pancreatitis, intrahepatic cholestasis, primary biliary cirrhosis, gallbladder and biliary duct tumors, drug-induced pancreatitis, gastroesophageal reflux, peptic ulcer disease, hepatitis, right-sided pneumonia, and appendicitis were excluded. Attack episode was defined as recurrence of disease after normal physical and laboratory findings. Patients were grouped according to the trimester in which the symptoms developed for the first time. Preterm delivery was defined as birth at <37 weeks of gestation. Low birth weight was defined as a birth weight of a live-born infant of less than 2500 gr. Patients’ age, week of gestation, parity, body mass index (BMI), initial diagnosis at admission, definitive diagnosis, attack episodes, treatment modalities, and obstetric and neonatal complications were evaluated.

### Statistical Analysis

Statistical analysis was performed using SPSS IBM 21 (IBM Co., Armonk, NY, USA). Descriptive analysis was performed including frequency, percentage, means, and standard deviation of the demographic features and disease history. The Shapiro-Wilk test was used to verify normality. The Kruskal-Wallis test was employed to analyze more than two variables in the study. P<0.05 was considered statistically significant.

## RESULTS

Overall, 59 women were diagnosed as having complicated gallstone disease in pregnancy. The demographic features of the patients are shown in Table 1. Thirteen (15.9%) of the patients presented in the first trimester, 25 (30.5%) patients in the second, and 21 (25.6%) patients presented in the third trimester. Table 1 summarizes the distribution of cases throughout pregnancy. Fifty-one (86.4%) of the 59 patients had one attack episode and 6 (10.2%) patients were admitted to hospital twice. Two (3.4%) patients had 3 attack episodes (Table 2).

Acute cholecystitis was the most commonly diagnosed complicated gallstone disease in pregnancy; 37 patients were diagnosed as having acute cholecystitis during pregnancy (Table 3).

ERCPs were performed in 4 patients and the procedure was conducted without radiation. Three of the 4 patients opted for laparoscopic surgery. Cholecystectomy was performed in 15 pregnant women; 9 (60%) patients underwent surgery during the second trimester, 1 (6.6%) patient had surgery in the first trimester, and the remainder (n=5) (33.3%) underwent surgery in the third trimester. Laparotomy was performed in 1 patient in the third trimester due to inadequate exploration during laparoscopy.

Perinatal outcomes are summarized in Table 4. Four of the 59 pregnant women had preterm delivery. Two of these had undergone surgery and the remainder were treated conservatively. One of 59 pregnant women had a missed abortion (8 weeks of gestation). That patient had been treated conservatively. Five infants had a low birth weight. No gross fetal anomalies were encountered in either the conservatively- or surgically-treated patients. One maternal death was encountered. This patient had severe acute pancreatitis.

## DISCUSSION

Although some pregnant patients experience uncomplicated cholelithiasis, an important proportion develop complicated gallstone disease defined as acute cholecystitis, choledocholithiasis, cholangitis, and gallstone pancreatitis^(3)^. It is widely understood that symptomatic gallstone disease in pregnancy is related with increased mortality risk for both the mother and fetus and may result in complications including spontaneous abortion, fetal abnormalities, preterm labor, and even death^(2,5,7)^. The management of symptomatic biliary disease during pregnancy has often been nonsurgical to avoid fetal and maternal harm^(10)^, but ironically, this results in a high rate of antepartum symptom recurrence^(2,8,9)^. Although laparoscopy is known to be safe in the second trimester, studies have reported the risk of preterm labor or spontaneous abortion with LC^(12,13)^.

There is agreement concerning the security of LC during the second trimester of pregnancy and some physicians also extend the indication to the first and third trimester^(12,14,15,16,17)^. Recent guidelines recommended LC during pregnancy for all symptomatic gallstone disease^(8,9)^ and laparoscopic treatment of acute abdominal disease has the same indications in pregnant and non-pregnant patients^(18)^. Studies reporting uterine injury during trocar placement, increased risk of preterm labor and spontaneous abortion with LC exist, even though they were performed in the second trimester^(12,13)^. The rate of preterm labor is 0-20% for LC^(19,20,21,22,23)^. Fetal mortality rates following LC range from 0 to 5.2%^(19,20,21,22,23)^. In our study, the rate of preterm labor was 3.4% and fetal mortality was not encountered after LC. Symptomatic gallstone disease has been related with increased mortality risk for the mother and fetus, besides the risk of interventions in pregnancy^(7)^. Complications including spontaneous abortion, fetal abnormalities, preterm labor, and even fetal and maternal death may occur. In our study, 1 missed abortion, 1 maternal death, 2 preterm deliveries, 3 low-birth-weight fetuses, and no fetal abnormalities were encountered in conservatively-treated patients. A case series in the literature reported that the most common reasons for biliary surgery during pregnancy were biliary colic in 70% of cases, followed by acute cholecystitis in 20%, choledocholithiasis in 7%, and acute biliary pancreatitis in the remaining 3% of cases^(14)^. In our study, acute cholecystitis was the most commonly diagnosed complicated gallbladder disease in pregnancy; 37 (62.7%) patients were diagnosed as having acute cholecystitis during pregnancy.

There is a very high rate of antepartum symptom recurrence with nonsurgical management of symptomatic biliary disease during pregnancy^(2,8,10)^. It was reported that non-operative management of symptomatic cholelithiasis in pregnancy was associated with frequent hospitalizations^(24,25)^. Recurrence rates after conservative treatment range between 40-92%. Recurrence of biliary pancreatitis was observed in 50% of patients after conservative treatment(24). A higher incidence of preterm labor for patients with conservative versus surgical treatment, with a clear relation with symptom recurrence was reported^(22)^. In our study, 6 (10.2%) patients had 2 attack episodes and 2 (3.4%) were admitted to hospital on three further occasions.

ERCP is an important therapeutic option in patients with biliary and pancreatic disease. ERCP is a very effective way to extract common bile duct stones^(11)^. In the literature, it was concluded that ERCP could be performed safely during pregnancy. On the other hand, a lower rate of term pregnancy, higher rate of preterm delivery, and low birth weight were more common when interventions were required during the first trimester^(26)^. In our study, ERCP was performed in 4 patients. Three out of the 4 patients chose LC.

We present our clinical experience because the diagnosis, course, and management of complicated gallstone disease is complicated. We aimed to determine the outcomes of pregnant patients. As a new thought, biliary tract screening by sonographic examination may be recommended before pregnancy, and LC prior to pregnancy may be suggested to prevent complications related to gallstones during gestation. It might be especially considered for pregnant patients with a high BMI and a history of multiple small stones in the gallbladder. However, further randomized controlled trials are required before this idea can be fully supported. There are some limitations of this study. This study had a retrospective design and the patient population was small; further studies with greater patient populations will highlight possible missing comments.

## Figures and Tables

**Table 1:**
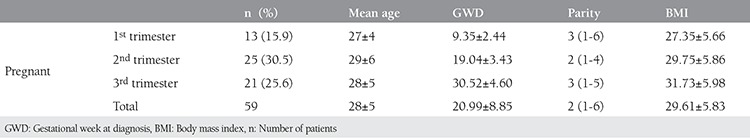
Demographic features of the patients and distribution of cases throughout pregnancy

**Table 2:**
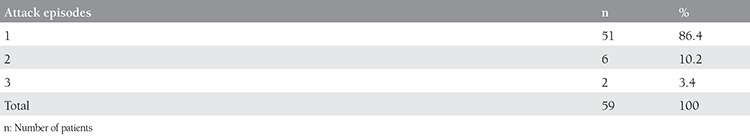
Attack episodes

**Table 3:**
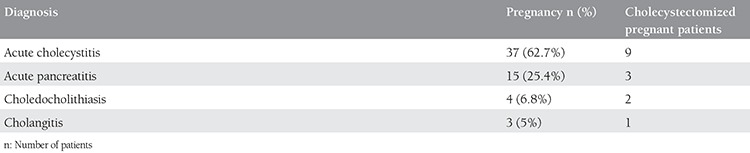
Distribution of patients according to diagnosis and surgical intervention

**Table 4:**
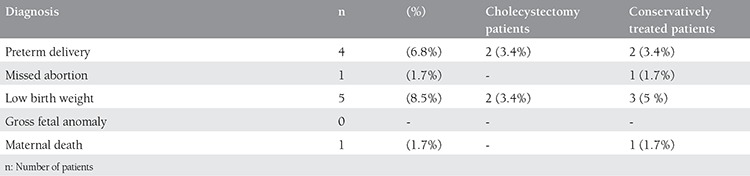
Perinatal outcomes of the cases
